# Supplementary material for: Associations of Food and Nutrient Intake with Serum Hepcidin and the Risk of Gestational Iron-Deficiency Anemia among Pregnant Women: A Population-Based Study
Source: Nutrients. 2021 Oct 3;13(10):3501. doi: 10.3390/nu13103501 (PMC8537751; doi:10.3390/nu13103501)
Supplement: Supplementary file 1 [file nutrients-13-03501-s001.zip › nutrients-1383968-Supplementary.pdf]

Supplementary Table S1. Food items and food groups included in the multiple linear regression analysis of gestational serum hepcidin levels

Food group		Food items	
1.	Rice and rice porridge	1.	Rice and grains.
		2.	Rice porridge.
2.	Noodles and related products	3.	Noodles and related products.
3.	Breakfast cereals, oats, and related products	4.	Breakfast cereals, oats, and related products.
4.	Bread and its products	5.	Bread its products.
5.	Chinese dim sum and related foods	6.	Chinese savory cakes.
		7.	Chinese buns and rolls.
		8.	Salty steamed buns.
		9.	Dumplings.
6.	Sweet buns, bean buns, and related products	10.	Sweet buns; red beans.
		11.	Mung beans, black eye beans, speckled kidney beans, and other seed beans and their products.
7.	Savory and sweet glutinous rice products	12.	Glutinous rice.
		13.	Glutinous rice desserts.
8.	Roots and tubers	14.	Root and tubers.
9.	Eggs	15.	Eggs.
10.	Dairy products	16.	Fresh milk, milk powder.
		17.	Flavored milk.
		18.	Yogurt.
		19.	Cheese.
11.	Non-dairy milk products	20.	Soy milk, honey/soy milk, tofu pudding and other soy milk drinks or products.
		21.	Rice milk.
12.	Light-colored vegetables	22.	Light-colored vegetables.
13.	Dark leafy vegetable	23.	Dark-colored vegetables
14.	Gourds, shoots, and root vegetables	24.	Carrots.
		25.	Root vegetables.
		26	Bamboo shoot.
		27.	Gourds.
15.	Mushrooms and related products	28.	Mushrooms.
16.	Legumes	29.	Beans, legumes.
17.	Marine vegetables	30.	Sea vegetables.
		31.	Kelp.
18.	Pickled vegetables	32.	Pickled vegetables.
19.	Herbs and spices	33.	Herbs and spices.
20.	Total vegetables		Total vegetables
21.	Soybean products	34.	Gluten and gluten sausages.
		35.	General soy products.
		36.	Soy-fermented foods.
22.	Nuts and seeds	37.	Nuts and seeds.
23.	Fresh fruits	38.	Fresh fruits.
24.	100% pure fruit juice or fruit/vegetable juice	39.	100% pure fruit juice or fruit and vegetable juice.
		40.	Commercially available sugary vegetable juices.
25.	Canned fruits, dried fruits, and jam	41.	Canned fruits.
		42.	Dehydrated fruits, pickled fruits, candied fruits.

	43. Jam.
26. Fish, shellfish, and seafood	44. Fish (including all fish).<br>45. Aquatic products eaten with bones or shell.<br>46. Edible sea snails and mollusks.<br>47. Other seafood except fish and mollusks.
27. Omega-3-rich fish	48. Deep-sea (high ω-3) fish.
28. Roe and processed seafood products	49. Roe of marine animals.<br>50. Processed seafood products.
29. Poultry meat	51. Poultry meat.
30. Lean livestock meat	52. Lean livestock meat.
31. Medium-fat livestock meat	53. Medium-fat livestock meat.
32. Liver, organs, and blood products	54. Liver.<br>55. Other internal organs besides the liver.<br>56. Poultry and livestock blood.<br>57. Other parts of poultry and livestock.
33. Processed meat products	58. Processed meat products.<br>59. Processed meat paste products.
